# The Effect of 2′-Fucosyllactose on Gut Health in Aged Mice

**DOI:** 10.3390/foods14244184

**Published:** 2025-12-05

**Authors:** Songsong Jiang, Yang Li, Tingting Luo, Yutong Huang, Huilian Che, Jinzhu Pang, Xiangren Meng

**Affiliations:** 1 Key Laboratory of Chinese Cuisine Intangible Cultural Heritage Technology Inheritance, Ministry of Culture and Tourism, College of Tourism and Culinary Science, Yangzhou University, Yangzhou 225127, China; 13856804665@163.com (T.L.); 19825302332@163.com (Y.H.); xrmeng@yzu.edu.cn (X.M.); 2 Mengniu Institute of Nutrition Science, Global R&D Innovation Center, Inner Mongolia Mengniu Dairy (Group) Co., Ltd., Beijing 101107, China; liyang117112@mengniu.cn; 3 Beijing Laboratory for Food Quality and Safety, College of Food Science and Nutritional Engineering, China Agricultural University, Beijing 100083, China; chehuilian@cau.edu.cn

**Keywords:** 2′-fucosyllactose, gut health, 16S rRNA, gut microbiota, aged mice

## Abstract

This study aimed to explore the effect of 2′-Fucosyllactose (2′-FL) on the gut health of aged mice. The results revealed that 2′-FL intervention effectively improved the intestinal permeability and reduced the serum diamine oxidase (DAO) level in aged mice (*p* < 0.05); in addition, 2′-FL increased the IgA level and decreased the IgG level (*p* < 0.05). Meanwhile, 2′-FL reduced the serum levels of IL-6, IL-1β, TNF-α, and IFN-γ (*p* < 0.05). Histopathological analysis indicated that 2′-FL successfully reversed intestinal damage in the jejunum and colon. Additionally, 2′-FL increased the expression of the tight-junction proteins ZO-1 and Claudin-1 both at mRNA and protein levels (*p* < 0.05), and also down-regulated the expression of pro-inflammatory factors (IL-6, IL-1β) (*p* < 0.05), and decreased aging-related markers P16^INK4α^ and P21^Cip1^. Furthermore, 16S rRNA results showed that 2′-FL increased the relative abundance of beneficial bacteria in the gut, such as *Lachnospiraceae*_UCG-006, norank_f__*Muribaculaceae* and *Lachnospiraceae_*NK4A136_group. In conclusion, 2′-FL effectively improved the intestinal immune health of the aged mice and provided a theoretical basis for its application as a functional component in the treatment of intestinal diseases.

## 1. Introduction

Human society is facing significant challenges due to the accelerated aging of the population, with gut health issues in older people being particularly prominent [1]. According to the results of China’s seventh national census, the population aged 60 and above has reached 260 million, accounting for 18.7% of the total population. Among them, 190 million people (equivalent to 13.5%) are aged 65 and above [2]. Intestinal diseases amongst older people are becoming increasingly severe. Overall, the average prevalence rate of constipation worldwide was estimated to be 16%, while the prevalence rate of constipation among adults aged 60 to 110 was 33.5% [3]. As individuals age, gut function declines markedly, not only resulting in weakened barrier integrity and increased intestinal permeability but also leading to dysbiosis and the persistent activation of chronic low-grade inflammation [4]. These alterations are closely associated with several age-related diseases, including chronic inflammation, neurodegenerative disorders, cardiovascular diseases, and metabolic disturbances [5]. The gut microbiome, a crucial ecosystem for host nutrition, immune regulation, and disease defense, undergoes complex changes in composition and function due to various factors such as age, diet, and environment [6]. Older people typically experience a deficiency in beneficial bacteria (such as *Bifidobacteria* and Butyrate-producing bacteria), and this reduced microbial diversity, along with lower levels of beneficial metabolic products, adversely affects mucosal barrier repair and immune homeostasis, thereby fostering inflammation and the progression of chronic diseases [1].

In recent years, human milk oligosaccharides (HMOs) have gained significant attention for their potential as functional dietary supplements. HMOs are the third most abundant component of breast milk, consisting of various small molecules with distinct structures [7]. Among them, 2′-fucosyllactose (2′-FL) is present at relatively high concentrations and exhibits unique biological activities [8]. Both 2′-FL and related HMOs serve as selective substrates for beneficial gut microbes, such as *Bifidobacteria* and *Akkermansia*, thereby enhancing microbiome diversity [9]. Additionally, they regulate immune function, promote intestinal barrier repair, and exhibit anti-pathogenic adhesion and anti-inflammatory effects through multiple mechanisms [10]. Notably, recent clinical and animal studies have shown that 2′-FL can stimulate the proliferation of *Bifidobacteria* in old age, improve metabolic status, and preliminarily suggest its potential to enhance immune responses and cognitive function. The underlying multi-omics mechanisms involve the gut–brain–immune axis, leading to systemic health benefits [11].

Despite abundant evidence demonstrating the essential role of human milk oligosaccharides (HMOs), particularly 2′-fucosyllactose (2′-FL), in facilitating gut microbiome colonization and immune maturation in infants, research on their utilization and molecular mechanisms in older people still remains rare. Considering the vulnerability of gut microbiota in older people, the prevalent presence of chronic inflammation, and the limited effectiveness of conventional interventions, investigating the impact of 2′-FL on gut microbiota and immune health in older people presents significant theoretical and practical promise. This study employed aged C57BL/6J mice as a model to methodically assess the effects of 2′-FL intervention on intestinal barrier function, microbiota composition, and immune responses, and this study will furnish scientific substantiation for the advancement of innovative functional foods and gut microbiota intervention strategies tailored for older people.

## 2. Materials and Methods

### 2.1. Materials

**Figure 1:**
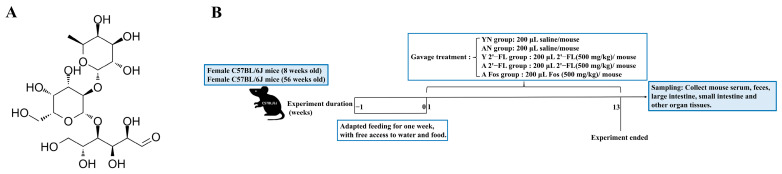
Animal experimental procedures. (**A**) Structural diagram of 2′-FL. (**B**) Experimental flowchart. YN, AN, Y 2′-FL, A 2′-FL, and A FOS represent The Young Mice Negative Control Group (YN), The Aged Mice Negative Control Group (AN), The Young Mice with 2′-FL Intervention Group (Y 2′-FL), The Aged Mice with 2′-FL Intervention Group (A 2′-FL), and The Aged Mice with FOS Intervention Group (A FOS), respectively. (Figure 1A Source: ChemSource website, https://www.chemsrc.com/cas/41263-94-9_21752.html#ebiemingDiv, accessed on 29 November 2025).

2′-Fucosyllactose was derived from Synaura Biotechnology (Shanghai) Co., Ltd. (Shanghai, China), a subsidiary of Mengniu Dairy (Group) Co., Ltd. (Beijing, China), the structural diagram of 2′-FL was shown in Figure 1A. Fructooligosaccharides (FOS) were purchased from Liuzhou Hongxu Biotechnology Co., Ltd. (commissioned by Chongqing Meiyitian Trading Co., Ltd.) (Liuzhou, China). Dextran (FITC-labeled dextran, 4 kDa, Sigma-Aldrich) was purchased from Sigma-Aldrich (St. Louis, MI, USA). A BCA protein concentration assay kit was purchased from Nanjing Norvizan Biotechnology Co., Ltd. (Nanjing, China). Goat anti-mouse IgA-HRP and Goat anti-mouse IgG-HRP were purchased from GeneTex (Irvine, CA, USA). Protein Marker was purchased from Thermo Fisher Scientific (Waltham, MA, USA). IL-1β, IL-6, INF-γ, TNF-α, IgA, IgG, and Diamine Oxidase (DAO) ELISA kits were purchased from R&D Systems (Minneapolis, MN, USA). RPMI-1640 medium was purchased from Gibco (USA); 4% paraformaldehyde and 1× Phosphate-Buffered Saline (PBS) were purchased from Wuhan Saier Biological Technology Co., Ltd. (Wuhan, China). ECL reaction solution was purchased from BIO-RAD (Hercules, CA, USA). Rabbit anti-Claudin-1 antibody was purchased from Wuhan Sanying Biotechnology Co., Ltd. (Wuhan, China). Rabbit anti-ZO-1 antibody, Rabbit anti-IL-1β, and Rabbit anti-IL-6 antibodies were purchased from Affinity Biosciences (Cincinnati, OH, USA).

### 2.2. Animals

In total, 24 specific pathogen-free (SPF) C57BL/6J mice of the same batch (female, 8 weeks old) with a body weight of 20 ± 5 g and 36 SPF C57BL/6J mice of the same batch (female, 56 weeks old) with a body weight of 30 ± 5 g were purchased from Phenok Bio-Tech (Shanghai) Co., Ltd. (Shanghai, China; SCXK (Hu) 2023-0008). They were acclimated for 7 days under conditions of 22 ± 1 °C temperature, 55 ± 10% relative humidity, and a 12 h light/dark cycle. All mice were provided ad libitum access to a standard irradiated diet from the same batch, supplied by Jiangsu Collaborative Pharmaceutical Biotechnology Co., Ltd., to ensure a sterile feeding environment. The experimental protocol was approved by the Animal Ethics Committee of Yangzhou University (202503204) and conducted in accordance with established protocols.

### 2.3. Animal Experimental Procedures

After acclimatization, mice were divided to different groups randomly. Specifically, the aged mice were randomly divided into 3 groups (n = 12), and the young mice were randomly divided into 2 groups (n = 12): The Young Mice Negative Control Group (YN), The Aged Mice Negative Control Group (AN), The Young Mice with 2′-FL Intervention Group (Y 2′-FL), The Aged Mice with 2′-FL Intervention Group (A 2′-FL), and The Aged Mice with FOS Intervention Group (A FOS). The Y 2′-FL and A 2′-FL mice were orally administered 200 μL of 2′-FL (500 mg/kg) per day. The A FOS group received 200 μL of FOS (500 mg/kg) orally per day. YN and AN groups were orally administered with 200 μL of saline at the same time points for each mouse. During the experiment, the mental state, activity and diet status, fur glossiness, feces condition, and any deaths of the mice were observed daily. After 13 weeks, the mice were humanely euthanized and tissues were collected.

### 2.4. Indicator Detection

#### 2.4.1. Measurement of Fecal Water Content and pH Value

The initial mass of the wet feces (100 mg) was recorded, followed by drying in an oven at 80 °C for 2 h until a constant weight was achieved. The difference in fecal mass before and after drying was documented, with the process repeated thrice for each sample. Fresh feces (100 mg) were combined with sterile water in a 1:3 ratio, thoroughly vortexed, and centrifuged at 3000 rpm for 10 min at 4 °C. The supernatant was then collected after settling for 5–10 min for pH analysis, with each sample assessed three times. Fecal water content was determined using the formula below:

Fecal moisture content (%) = (Wet mass-Dry mass)/Wet mass × 100%(1)

#### 2.4.2. Intestinal Permeability Assessment

Intestinal permeability was assessed following the experimental protocol outlined by Qu et al. [12], with minor adjustments. Three mice were randomly chosen from each of the following groups: YN, AN, Y 2′-FL, A 2′-FL, and A FOS. The mice in the Y 2′-FL group received 200 μL of 2′-FL (500 mg/kg) orally, while those in the A FOS group were intragastrically administered 200 μL of FOS (500 mg/kg). Following a 4 h fasting period, all mice received 200 μL of FITC-labeled dextran dissolved in PBS (500 mg/kg). Blood was collected after 4 h and centrifuged at 3000× *g* for 10 min at 4 °C post 2 h rest, and the supernatant was extracted. Serum from each group was diluted in a 1:10 ratio with PBS, and fluorescence measurements were taken at excitation and emission wavelengths of 493 and 518 nm, respectively, using a SpectraMax M5 multifunctional microplate reader.

During the 13th week, blood samples were procured from the retro-orbital venous plexus of each mouse group, followed by a 2 h rest at 4 °C before serum extraction through centrifugation under 3000× *g* conditions. The serum DAO level in each group was quantified utilizing Indirect Enzyme-Linked Immunosorbent Assay (ELISA).

#### 2.4.3. Serum Antibody Detection

During the 13th week, blood samples were gathered from the retro-orbital venous plexus of each mouse group. Following a 2 h rest at 4 °C, the serum was collected through centrifugation at 3000× *g*. The serum samples from all mouse groups were analyzed for IgA and IgG antibody levels using Indirect Enzyme-Linked Immunosorbent Assay (ELISA).

#### 2.4.4. Detection of Cytokine Levels

During the 13th week, serum samples were collected from mice, and the levels of cytokines (IL-1β, IL-6, IFN-γ, TNF-α) in the serum samples were assessed using an ELISA kit.

#### 2.4.5. Histological Analysis of Tissues

After euthanasia of mice, intestinal tissues were fixed in 4% paraformaldehyde, and processed, followed by paraffin embedding, and sectioned at 5–10 μm. Sections were stained with hematoxylin and eosin (H&E) following standard protocols, dehydrated through graded ethanol, cleared in xylene, and mounted with resin. Slides were examined by light microscopy and imaged.

#### 2.4.6. RT-qPCR

RT-qPCR was performed following Sun et al. [13], with appropriate modifications, to detect the levels of tight junction proteins ZO-1 and Claudin-1, inflammatory factors IL-1β and IL-6, and key aging factors P16^INK4α^ and P21^Cip1^ in the mouse small intestine. Total RNA extraction from tissue samples: Tissues weighing 30–40 mg were homogenized in 1 mL of Trizol Reagent. Following centrifugation and separation of the aqueous phase, the extracted material was combined with an equal volume of isopropanol in a new centrifuge tube, centrifuged to remove the supernatant, and washed with 1 mL of 75% ethanol before drying. RNA concentration and purity were determined using a Denovix UV-Vis spectrophotometer, adjusting the concentration to 100 ng/μL. Then, standard gene reverse transcription was conducted using the Cwbio RT KIT as per the manufacturer’s guidelines. Quantitative PCR: Primer sequences are listed in Table 1. A mixture comprising 10 μL of Hieff™ qPCR SYBR^®^ Green Master Mix (No Rox), 1 μL of Forward Primer, 1 μL of Reverse Primer, and 100 ng of template DNA was prepared, supplemented with ddH2O up to a total volume of 20 μL. Quantitative PCR analysis was executed on the Bio-Rad CFX system, featuring a thermal cycling profile with an initial denaturation at 95 °C for 5 min, followed by 44 cycles of denaturation at 95 °C for 15 s, annealing at 55 °C for 30 s, and extension at 72 °C for 20 s. Subsequent data analysis involved relative quantification utilizing the 2^−ΔΔCT^ method with GAPDH as the internal control.

#### 2.4.7. Western Blot

Western blot was conducted according to the method of Wang et al. [14] with appropriate modifications. The total protein content of tissues and cells was extracted using RIPA lysis buffer containing PMSF. After centrifugation, the supernatant containing the extracted total protein was stored at −80 °C. A protein standard solution was prepared at a concentration of 25 mg/mL, which was then diluted to a final concentration of 0.5 mg/mL and quantified using a BCA assay kit. Adjusting the protein concentration to 1 μg/μL, each sample was boiled and stored at −80 °C for future use. An 8–12% SDS gel was utilized for electrophoretic separation. Subsequently, the proteins were transferred to a PVDF membrane, followed by immediate immersion in pre-prepared TBST wash solution for 1–2 min to clear the transfer liquid, and blocking with 5% skim milk for 1 h. After blocking, the milk was removed, and the primary antibody was applied, incubating for 2 h. Sequentially, the diluted HRP-conjugated secondary antibody was added, allowing a 2 h incubation at room temperature. Post-incubation, the secondary antibody solution was discarded, and the membranes were washed on a shaker thrice for 10 min each with TBST. Once washed, the membranes were prepared for further procedures. Subsequent to a 1 min development with the ECL reaction solution, band imaging was performed using the Gel Doc 2000 imaging system.

### 2.5. 16S rRNA Gene Sequencing

16S rRNA gene sequencing was performed to analyze the composition of the intestinal microbiota referenced to the method by Katiraei et al. [15]. Cecum samples were collected from five mouse groups, promptly frozen in liquid nitrogen, and stored at −80 °C. Genomic DNA was extracted using a phenol–chloroform–isoamyl alcohol (25:24:1) (Invitrogen) (Carlsbad, CA, USA) method from the cecum samples, followed by isopropanol precipitation, 70% ethanol washing, and subsequent DNA isolation. The DNA quantity was assessed using a Nanodrop 5000 spectrophotometer. The V3-V4 region of the 16S rRNA gene was amplified and sequenced utilizing the Illumina NovaSeq platform (Illumina NovaSeq) (San Diego, CA, USA). A forward primer (5′-GTGCCAGCMGCCGCGGTAA-3′) and reverse primer (5′-GGACTACHVGGGTWTCTAAT-3′) were employed. The bioinformatics analysis of the sequencing data consisted of filtering and aligning the raw data to produce high-quality sequences. Subsequently, these sequences were clustered into Operational Taxonomic Units (OTUs) with a sequence similarity threshold of 97%.

### 2.6. Data Analysis

The data was presented as the mean ± standard deviation. Statistical analysis was conducted with GraphPad Prism 8.0.2 (GraphPad Software, Inc.) and SPSS 19.0 (SPSS Software, Inc.). Data were analyzed using the one-way ANOVA followed by Duncan’s multiple range test. Differences among groups were considered statistically significant at *p* < 0.05, and all data satisfied the required statistical assumptions. Each experiment was repeated at least three times.

## 3. Results

### 3.1. Effects of 2′-FL on Body Weight and Physiological Status of Mice in Each Group

**Figure 2:**
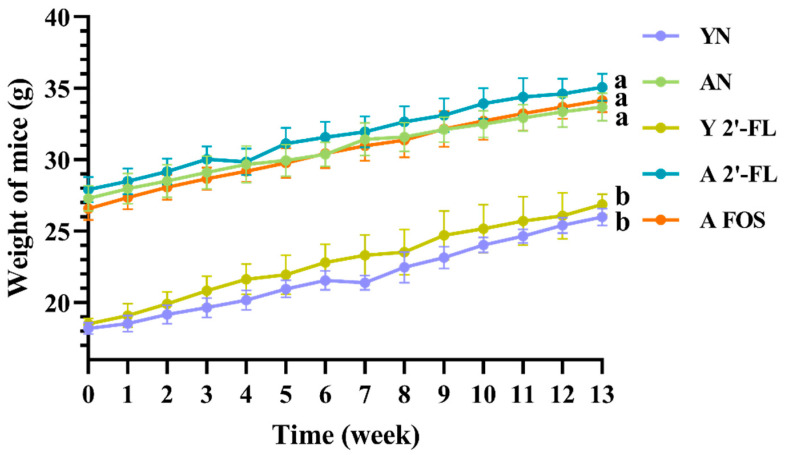
Graph of mouse body weight changes. YN, AN, Y 2′-FL, A 2′-FL, and A FOS represent The Young Mice Negative Control Group (YN), The Aged Mice Negative Control Group (AN), The Young Mice with 2′-FL Intervention Group (Y 2′-FL), The Aged Mice with 2′-FL Intervention Group (A 2′-FL), and The Aged Mice with FOS Intervention Group (A FOS), respectively. Different letters indicate significant differences (*p* < 0.05), n = 12.

As depicted in Figure 2, no significant differences in body weight were observed among mice in different groups at various time points (weeks 0–13). Both the YN and AN groups displayed consistent and normal growth patterns in body weight. Mice in the Y 2′-FL, A 2′-FL, and A FOS groups did not exhibit notable weight loss or anomalies, with all mice showing positive weight gain, suggesting that oral administration of 2′-FL and FOS did not have detrimental effects on their growth. Throughout the experiment, the mice in each group maintained normal mental states, fur quality, activity levels, eating habits, and overall health, without any signs of diarrhea or abnormal behavior.

### 3.2. Effects of 2′-FL on Fecal Moisture and pH Values in Mice of Each Group

**Figure 3:**
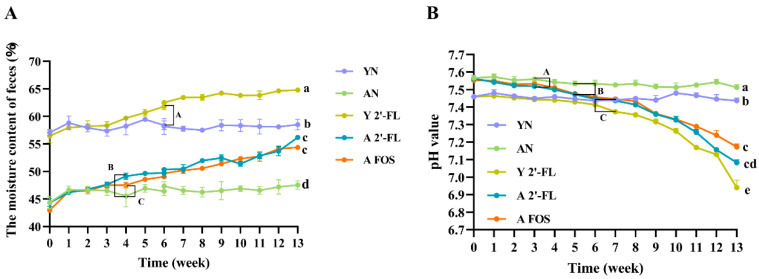
Graph showing the changes in moisture content and pH value of mouse feces. (**A**) Graph showing the changes in moisture content of mouse feces. (**B**) Graph showing the changes in pH value of mouse feces. YN, AN, Y 2′-FL, A 2′-FL, and A FOS represent The Young Mice Negative Control Group (YN), The Aged Mice Negative Control Group (AN), The Young Mice with 2′-FL Intervention Group (Y 2′-FL), The Aged Mice with 2′-FL Intervention Group (A 2′-FL), and The Aged Mice with FOS Intervention Group (A FOS), respectively. Different lowercase letters indicate significant differences among the groups at the end of the experiment, while different uppercase letters indicate significant differences between groups during the experimental period (*p* < 0.05). n = 12.

Mouse fecal water content serves as an indicator of intestinal microenvironmental changes to some extent [16]. As shown in Figure 3A, following a 3-week 2′-FL intervention, the Y 2′-FL group exhibited a notable increase in fecal water content compared to the YN group. Subsequently, after 6 weeks of intervention, the fecal water content showed a significant increase (*p* < 0.05). Following a four-week treatment with 2′-FL and FOS, both the A 2′-FL and A FOS groups exhibited a notable increase in fecal water content (*p* < 0.05), particularly in the A 2′-FL group. Notably, in aged mice, prolonged intervention led to partial constipation relief in some individuals, which might be associated with the observed rise in fecal water content. Collectively, these results indicated that 2′-FL effectively enhanced fecal water content in aged mice.

The pH of mouse feces correlates with the proliferation of beneficial intestinal bacteria, potentially benefiting intestinal health [17]. In Figure 3B, the AN group mice exhibited higher fecal pH values compared to the YN group, likely due to age-related declines in microbial diversity, resulting in elevated pH level and a reduction in SCFA-producing bacteria [18]. Following a 4-week 2′-FL intervention, the Y 2′-FL group mice demonstrated a decreased in fecal pH. After 7 weeks of intervention, a significant reduction in fecal pH level was observed (*p* < 0.05). In contrast to the AN group, the A 2′-FL group mice showed a significant decrease in fecal pH after just 3 weeks of 2′-FL intervention (*p* < 0.05). Similarly, the A FOS group mice exhibited significantly lower fecal pH value compared to the AN group after 5 weeks of Fos intervention (*p* < 0.05). These results indicated that 2′-FL effectively reduced fecal pH in aged mice.

### 3.3. Effects of 2′-FL on Intestinal Permeability in Mice of Each Group

**Figure 4:**
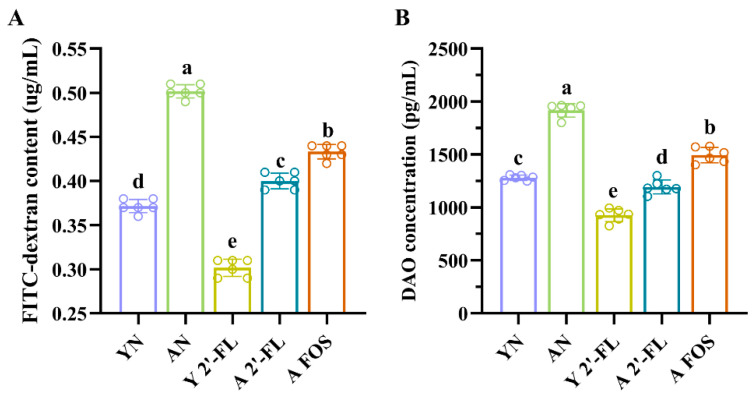
Intestinal permeability and DAO level of mice in different treatment groups. (**A**) Changes in intestinal permeability. (**B**) DAO level. YN, AN, Y 2′-FL, A 2′-FL, and A FOS represent The Young Mice Negative Control Group (YN), The Aged Mice Negative Control Group (AN), The Young Mice with 2′-FL Intervention Group (Y 2′-FL), The Aged Mice with 2′-FL Intervention Group (A 2′-FL), and The Aged Mice with FOS Intervention Group (A FOS), respectively. Different letters indicate significant differences (*p* < 0.05), n = 6.

Following oral administration, FITC-labeled dextran is absorbed across the intestinal wall into the bloodstream. The fluorescence intensity of serum FITC–dextran positively correlates with intestinal permeability and serves as a quantitative marker for both gut barrier integrity and mucosal function [19]. As shown in Figure 4A, compared to the AN group, the Y 2′-FL group exhibited a significant reduction in intestinal permeability, as evidenced by a marked decrease in serum FITC–dextran level (*p* < 0.05). These results suggested that 2′-FL significantly reduced intestinal permeability in Y 2′-FL mice (*p* < 0.05). Additionally, compared to the AN group, serum FITC–dextran concentrations were significantly lower (*p* < 0.05) following intervention with A 2′-FL and A FOS.

Serum DAO serves as an indirect biomarker of intestinal permeability and is widely used to assess intestinal integrity [20]. As shown in Figure 4B, compared to the YN group, DAO level in the AN group was significantly elevated (*p* < 0.05). In contrast, DAO level was significantly reduced in the Y 2′-FL group compared to the YN group (*p* < 0.05). Similarly, DAO level in the A 2′-FL group was significantly lower than those in the AN group (*p* < 0.05). Compared to the AN group, DAO level in the intervention groups was significantly reduced (*p* < 0.05). This study showed that both 2′-FL and FOS could significantly reduce intestinal permeability; 2′-FL tended to show stronger effects than FOS, though direct differences were not significant.

### 3.4. Effects of 2′-FL on Antibody Levels in Mice of Each Group

**Figure 5:**
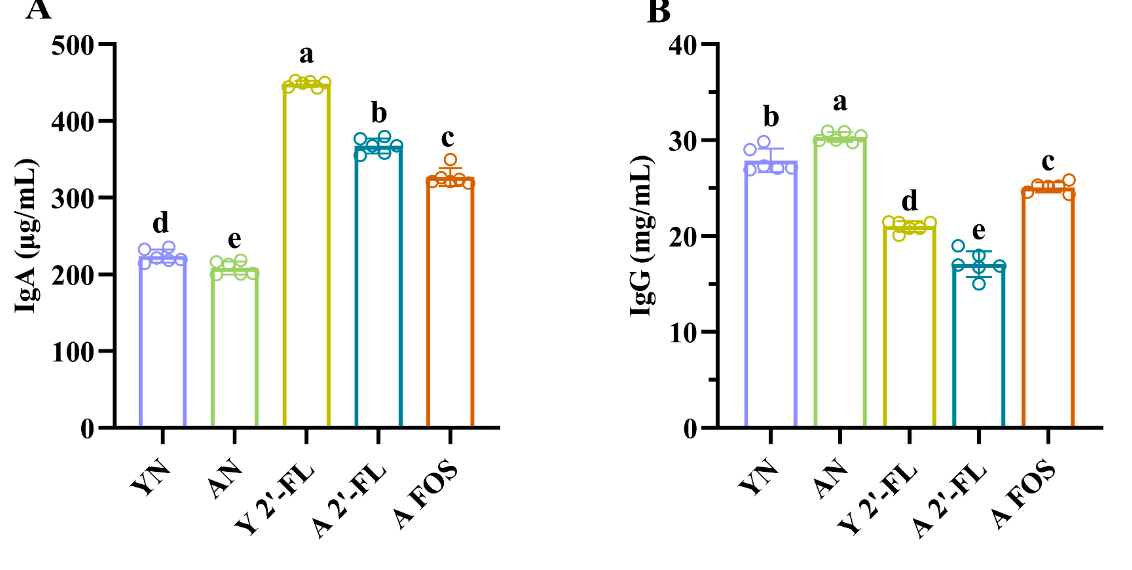
Antibody levels of IgA and IgG in mice of different treatment groups. (**A**) IgA level. (**B**) IgG level. YN, AN, Y 2′-FL, A 2′-FL, and A FOS represent The Young Mice Negative Control Group (YN), The Aged Mice Negative Control Group (AN), The Young Mice with 2′-FL Intervention Group (Y 2′-FL), The Aged Mice with 2′-FL Intervention Group (A 2′-FL), and The Aged Mice with FOS Intervention Group (A FOS), respectively. Different letters indicate significant differences (*p* < 0.05), n = 6.

IgA and IgG levels serve as key indicators for assessing the intestinal mucosal immune status, gut microbiota balance, and the degree of systemic immune aging in mice [21]. In this study, IgA and IgG antibody levels were measured in mice using an indirect ELISA method. As shown in Figure 5A, IgA levels in the AN group were significantly lower than those in the YN group (*p* < 0.05). In contrast, IgA levels in the Y 2′-FL group were significantly higher than those in the YN group (*p* < 0.05). Similarly, IgA levels in the A 2′-FL and A FOS groups were significantly higher than those in the AN group (*p* < 0.05). Furthermore, IgG level in the AN group was significantly higher than in the YN group (*p* < 0.05) (Figure 5B). Compared to the YN group, serum IgG level was significantly reduced in the Y 2′-FL group (*p* < 0.05), and IgG levels in the A 2′-FL and A FOS groups were significantly lower than those in the AN group (*p* < 0.05), particularly in the E 2′-FL group.

### 3.5. Effects of 2′-FL on Cytokine Levels in Mice of Each Group

**Figure 6:**
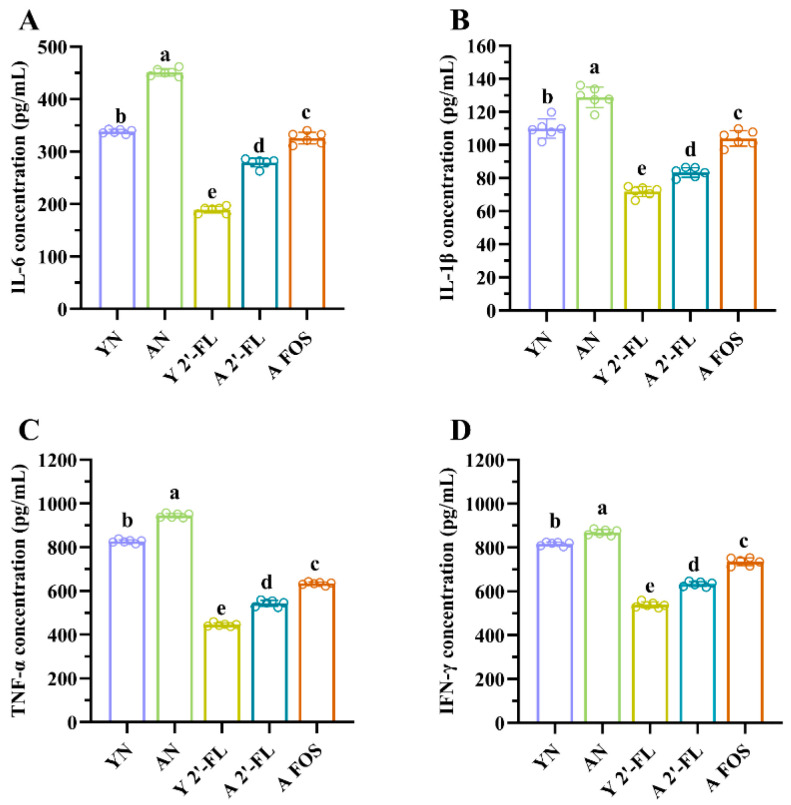
Levels of inflammatory cytokines in mice of different treatment groups. (**A**) IL-6 level. (**B**) IL-1β level. (**C**) TNF-α level. (**D**) INF-γ level. YN, AN, Y 2′-FL, A 2′-FL, and A FOS represent The Young Mice Negative Control Group (YN), The Aged Mice Negative Control Group (AN), The Young Mice with 2′-FL Intervention Group (Y 2′-FL), The Aged Mice with 2′-FL Intervention Group (A 2′-FL), and The Aged Mice with FOS Intervention Group (A FOS), respectively. Different letters indicate significant differences (*p* < 0.05), n = 6.

Inflammatory cytokines play an integral role in gut and immune health, demonstrating a strong association with age-related chronic inflammation [22]. This study initially quantified serum levels of various cytokines in mice (Figure 6): IL-6, IL-1β, TNF-α, and IFN-γ. Levels of inflammatory cytokines (IL-6, IL-1β, TNF-α, IFN-γ) were markedly lower in the YN group compared to the EN group (*p* < 0.05) (Figure 6A–D). The Y 2′-FL group exhibited significant reductions in inflammatory cytokine levels (IL-6, IL-1β, TNF-α, IFN-γ) compared to the YN group (*p* < 0.05) (Figure 6A–D). Furthermore, the A 2′-FL and A FOS groups showed notably lower levels of inflammatory cytokines (IL-6, IL-1β, TNF-α, IFN-γ) than the AN group (*p* < 0.05) (Figure 6A–D). This study highlighted that both 2′-FL and FOS could significantly reduce the levels of inflammatory factors, 2′-FL tended to show stronger effects than FOS, though direct differences were not significant.

### 3.6. Tissue Pathological Analysis

**Figure 7:**
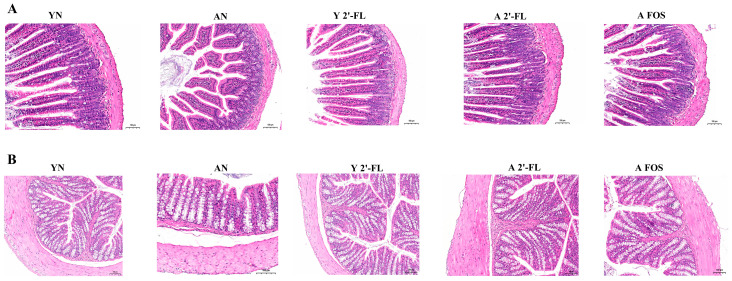
Histopathological analysis of mouse tissues. (**A**) HE staining of the jejunum. (**B**) HE staining of the colon. YN, AN, Y 2′-FL, A 2′-FL, and A FOS represent The Young Mice Negative Control Group (YN), The Aged Mice Negative Control Group (AN), The Young Mice with 2′-FL Intervention Group (Y 2′-FL), The Aged Mice with 2′-FL Intervention Group (A 2′-FL), and The Aged Mice with FOS Intervention Group (A FOS), respectively.

Aging often leads to the degeneration of the mouse’s intestinal barrier, resulting in intestinal inflammation and damage to the mouse intestine [23]. The intestinal mucosa was stained with hematoxylin–eosin (HE) to evaluate the effect of 2′-FL on the jejunum intestines of aged mice. The HE staining of the small intestine revealed that the AN group, in comparison to the YN group, displayed villous atrophy, disorganized glandular arrangement, disrupted crypt structures, extensive shedding of intestinal epithelium, and infiltration of inflammatory cells into the lamina propria. Conversely, the Y 2′-FL group demonstrated significant restoration of intestinal villi height, a notable decrease in the number of inflammatory cells, and nearly complete return to normal levels compared to the YN group. In contrast to the AN group, the A 2′-FL group exhibited regeneration and substantial restoration of small intestinal villi, closer to a regular glandular arrangement, crypt restructuring, reduced inflammatory cell infiltration in the lamina propria, near-complete restoration of epithelial continuity, and effective suppression of intestinal inflammation in mice. The villi height within the small intestine tissue of the A FOS group showed a slight increase, indicating some improvement in intestinal inflammation. Nevertheless, certain crypt structures remained disordered, with partial infiltration of inflammatory cells (Figure 7A).

The hematoxylin and eosin (HE) staining of the mouse colon (Figure 7B) revealed that, in comparison to the YN group, the AN group exhibited disrupted structures in the small intestinal crypts. Crypts showed possible stem cell differentiation imbalance, depleted goblet cells, decreased mucous vesicle count. Conversely, the Y 2′-FL group displayed organized colonic crypt structures, no signs of dilation or fibrosis, evenly distributed goblet cells with purplish-red cytoplasmic vacuoles, and plentiful numbers. The A 2′-FL group, in contrast to the AN group, underwent glandular rearrangement, approaching the typical cryptic structure of the YN group, with a nearly restored density of mucous vesicles, and goblet cell regeneration. The A FOS group showed partial recovery of goblet cells, localized resolution of colonic inflammation, but the issue of glandular branching disorder remained unresolved.

### 3.7. Effects of 2′-FL on Gene Expression of Immune Aging and Oxidative Stress Factors in Mice of Each Group

**Figure 8:**
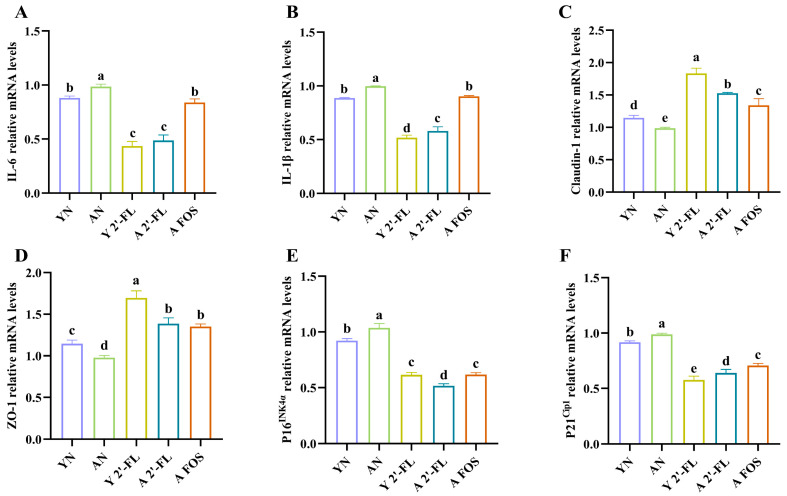
Analysis of immune aging and oxidative stress factor gene expression in various intestinal tissues of mice. (**A**) IL-6 gene expression. (**B**) IL-1β gene expression. (**C**) Claudin-1 gene expression. (**D**) ZO-1 gene expression. (**E**) P16^INK4α^ gene expression. (**F**) P21^Cip1^ gene expression. YN, AN, Y 2′-FL, A 2′-FL, and A FOS represent The Young Mice Negative Control Group (YN), The Aged Mice Negative Control Group (AN), The Young Mice with 2′-FL Intervention Group (Y 2′-FL), The Aged Mice with 2′-FL Intervention Group (A 2′-FL), and The Aged Mice with FOS Intervention Group (A FOS), respectively. Different letters indicate significant differences (*p* < 0.05), n = 3.

The impact of 2′-FL on cytokine expression levels in various intestinal tissues of naturally aging mice was assessed through quantitative analysis of gene expression. This evaluation included inflammatory factors (IL-6, IL-1β), tight junction proteins (Claudin-1, ZO-1), and key aging factors (P16^INK4α^, P21^Cip1^) using the qRT-PCR method, as depicted in Figure 8.

In the colon of the AN group, mRNA levels of IL-6 and IL-1β were significantly higher than those in the YN group (*p* < 0.05), indicating pronounced inflammation in aged mice (Figure 8A,B). Comparatively, the Y 2′-FL group exhibited a significant reduction in the expression of IL-6 and IL-1β inflammatory factors in the colon of young mice (*p* < 0.05) when contrasted with the YN group. Moreover, the A 2′-FL group demonstrated strong inhibition of IL-6 and IL-1β expression relative to the AN group (*p* < 0.05), confirming 2′-FL-mediated anti-inflammatory effects in aged mice. Although the A FOS group exhibited reduced expression levels of IL-6 and IL-1β relative to the AN group, the difference did not reach statistical significance (*p* > 0.05). These findings align with the analysis of IL-6 and IL-1β inflammatory cytokines in mice (Figure 6A,B).

Claudin-1 and ZO-1 are the main proteins related to the formation of tight junctions and are closely associated with the balance of barrier function in older people [24]. As shown in Figure 8C,D, the tight junction proteins Claudin-1 and ZO-1 in the jejunum of the AN group were significantly lower than those in the YN group (*p* < 0.05). Compared with the YN and AN groups, 2′-FL significantly increased the expression of tight junction proteins Claudin-1 and ZO-1 in the Y 2′-FL and A 2′-FL groups (*p* < 0.05); similarly, the expression level of tight junction protein Claudin-1 and ZO-1 in the A FOS group was significantly higher than that in the AN group (*p* < 0.05), but lower than that in the A 2′-FL group. This study indicated that both 2′-FL and FOS could significantly increase the levels of intestinal tight junction proteins; 2′-FL tended to show stronger effects than FOS, though direct differences were not significant.

P16^INK4α^ and P21^Cip1^, as key markers of intestinal aging, are closely related to the health of the aged intestinal tract and immune aging [25]. Compared with the YN group, the expression levels of the aging factors P16^INK4α^ and P21^Cip1^ in the AN group were significantly increased (*p* < 0.05) (Figure 8E,F). Specifically, the expression levels of the aging factors P16^INK4α^ and P21^Cip1^ in the Y 2′-FL group were significantly lower than that in the YN group (*p* < 0.05); the expression levels of the aging factors P16^INK4α^ and P21^Cip1^ in the A 2′-FL group were significantly lower than in the AN group (*p* < 0.05), and the aging factors P16^INK4α^ and P21^Cip1^ in the A FOS group were consistent with the change trend of the A 2′-FL group.

### 3.8. Effects of 2′-FL on the Expression of Intestinal-Related Inflammatory Factors and Tight Junction Proteins in Mice

**Figure 9:**
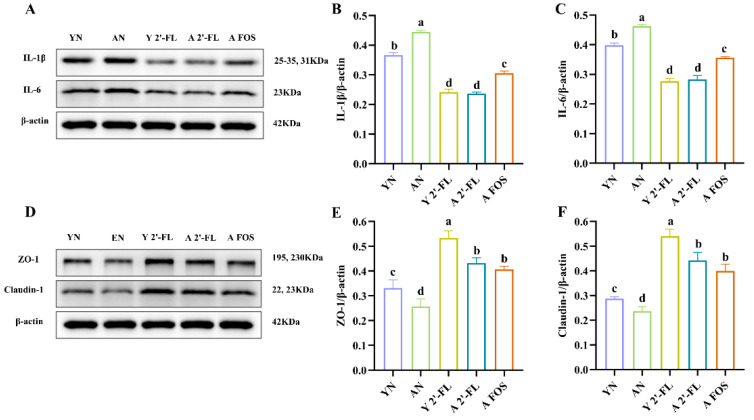
Analysis of expression of intestinal-related inflammatory factors and tight junction proteins in different treatment groups of mice. (**A**) Western blot analysis of expression of related inflammatory factors. (**B**) Quantitative analysis of IL-1β. (**C**) Quantitative analysis of IL-6. (**D**) Western blot analysis of expression of related tight junction proteins. (**E**) Quantitative analysis of ZO-1. (**F**) Quantitative analysis of Claudin-1. YN, AN, Y 2′-FL, A 2′-FL, and A FOS represent The Young Mice Negative Control Group (YN), The Aged Mice Negative Control Group (AN), The Young Mice with 2′-FL Intervention Group (Y 2′-FL), The Aged Mice with 2′-FL Intervention Group (A 2′-FL), and The Aged Mice with FOS Intervention Group (A FOS), respectively. Different letters indicate significant differences (*p* < 0.05), n = 3.

This study conducted a detailed analysis of colonic inflammatory factors IL-1β and IL-6, as well as small intestinal tight junction proteins ZO-1 and Claudin-1, utilizing Western blotting to investigate the impact of 2′-FL on the intestinal and immune health of aged mice. The findings are presented in Figure 9, encompassing sections A to C. In the colon, the levels of inflammatory factors IL-1β and IL-6 were markedly higher in the AN group than in the YN group (*p* < 0.05). Post 2′-FL administration, the Y 2′-FL and A 2′-FL groups exhibited a significant decrease in IL-1β and IL-6 levels compared to both the YN and AN groups (*p* < 0.05), while the A FOS group also demonstrated a noteworthy reduction in IL-1β and IL-6 expression (*p* < 0.05), especially in the A 2′-FL group. The protein expression patterns of IL-1β and IL-6 resonated with their gene expression profiles (Figure 8A,B).

In the YN group, there was a notable elevation in the expression of small intestinal tight junction proteins ZO-1 and Claudin-1 compared to the AN group (*p* < 0.05) (Figure 9D–F). Following 2′-FL intervention, the Y 2′-FL group exhibited a significant increase in the levels of ZO-1 and Claudin-1 (*p* < 0.05). Similarly, both the A 2′-FL and A FOS groups demonstrated a significant rise in ZO-1 and Claudin-1 expression levels compared to the AN group (*p* < 0.05), particularly in the A 2′-FL group. The protein expression patterns of ZO-1 and Claudin-1 were in agreement with their gene expression patterns (Figure 8C,D).

### 3.9. Effects of 2′-FL on the Gut Microbiota in Mice of Each Group

**Figure 10:**
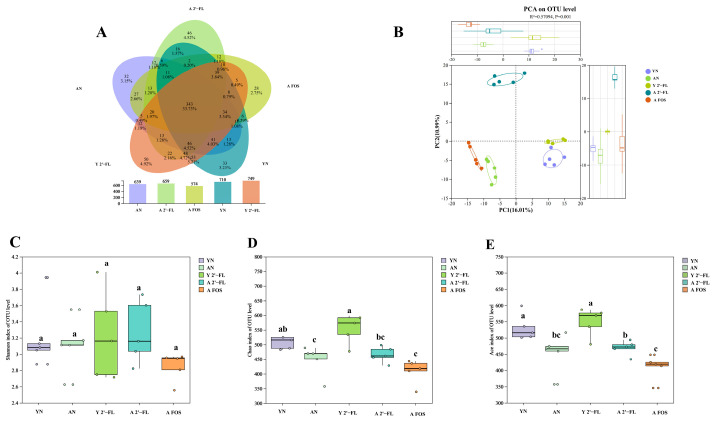
Analysis of intestinal microbiota in different treatment groups of mice. (**A**) Venn diagram among the groups. (**B**) PCA diagram. (**C**) Shannon index. (**D**) Chao index. (**E**) Ace index. YN, AN, Y 2′-FL, A 2′-FL, and A FOS represent The Young Mice Negative Control Group (YN), The Aged Mice Negative Control Group (AN), The Young Mice with 2′-FL Intervention Group (Y 2′-FL), The Aged Mice with 2′-FL Intervention Group (A 2′-FL), and The Aged Mice with FOS Intervention Group (A FOS), respectively. Different letters indicate significant differences (*p* < 0.05), n = 5.

The gut microbiota and the intestinal microecology generated by its habitat are closely intertwined with intestinal and immune health [26]. This study intervened in the mice’s gut microbiota with 2′-FL and performed sequencing of the 16S rRNA V3-V4 region to investigate the potential of 2′-FL in improving gut microbial balance and enhancing intestinal and immune health. All high-quality sequences were clustered at 97% identity, resulting in a total of 3331 operational taxonomic units (OTUs) across the five sample groups, with 639, 659, 574, 710, and 749 OTUs identified in the AN, A 2′-FL, A FOS, YN, and Y 2′-FLgroups, respectively. The Venn diagram illustrated shared and unique OTUs within the gut microbiota of the five mouse groups (Figure 10A). Principal Coordinate Analysis (PCA) was applied to depict the similarities and dissimilarities among microbial communities in various sample groups. As illustrated in Figure 10B, there were small clustering distances within each group, suggesting significant separation between different treatment groups and distinctive differences in microbial communities among the samples. Further assessment of α-diversity in the mouse gut microbiota was conducted to evaluate microbial community richness and diversity. Common indices, including ACE and Chao for community richness and Shannon index for community diversity, were employed to characterize microbial diversity. Figure 10C–E show that the Shannon index in the AN group exceeded that in the YN group (*p* > 0.05), while the Chao and Ace indices were notably higher than those in the AN group (*p* < 0.05), indicating that gut microbial community richness were significantly lower in aged mice compared to young mice. Post-treatment with 2′-FL led to a notable increase in the Shannon, Chao, and Ace indices in the Y 2′-FL group compared to the YN group. Similarly, the Shannon index in the A 2′-FL group was higher than that in the AN group (*p* > 0.05), with the Chao and Ace indices significantly higher than in the AN group (*p* > 0.05), contrasting the trend in the A FOS group. The study findings suggested that 2′-FL could effectively enhance gut microbial community diversity and richness in aged mice.

**Figure 11:**
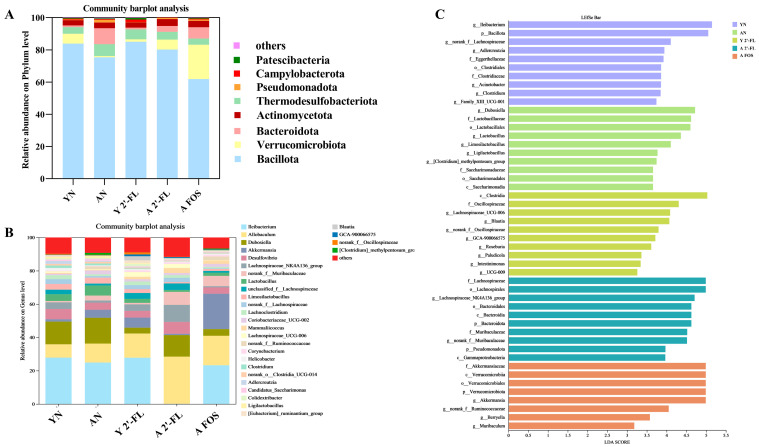
Analysis of intestinal microbiota in different treatment groups of mice. (**A**) Differences at the phylum level. (**B**) Differences at the genus level. (**C**) LDA score plot. YN, AN, Y 2′-FL, A 2′-FL, and A FOS represent The Young Mice Negative Control Group (YN), The Aged Mice Negative Control Group (AN), The Young Mice with 2′-FL Intervention Group (Y 2′-FL), The Aged Mice with 2′-FL Intervention Group (A 2′-FL), and The Aged Mice with FOS Intervention Group (A FOS), respectively. Different letters indicate significant differences (*p* < 0.05), n = 5.

**Figure 12:**
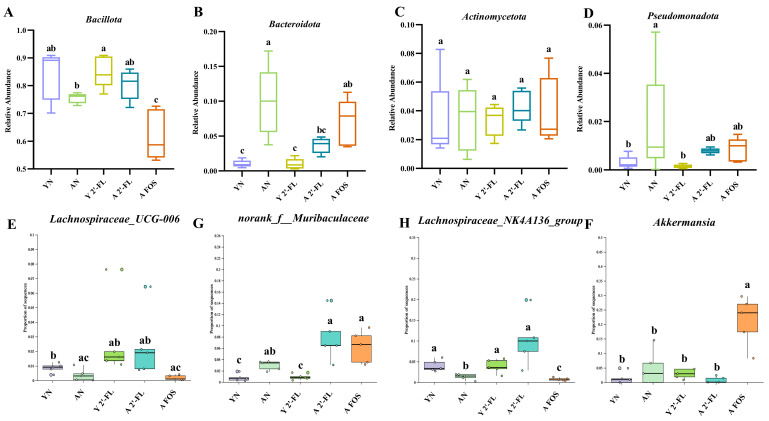
Analysis of intestinal microbiota in different treatment groups of mice. (**A**–**D**) Relative abundances of microbiota with significant differences at the phylum level. (**E**–**H**) Sequence proportions of microbiota with significant differences at the genus level. YN, AN, Y 2′-FL, A 2′-FL, and A FOS represent The Young Mice Negative Control Group (YN), The Aged Mice Negative Control Group (AN), The Young Mice with 2′-FL Intervention Group (Y 2′-FL), The Aged Mice with 2′-FL Intervention Group (A 2′-FL), and The Aged Mice with FOS Intervention Group (A FOS), respectively. Different letters indicate significant differences (*p* < 0.05), n = 5.

Upon analyzing the gut microbiota differences among the mouse treatment groups, Figure 11A,B illustrate the relative abundance of annotated species at the phylum and genus levels of the mouse microbiota. Figure 11A demonstrates that *Bacillota (formerly Firmicutes)* and *Bacteroidota* are the dominant phyla in all treatment groups, aligning with their prominent presence in healthy mouse gut microbiota. Additionally, smaller proportions of *Verrucomicrobiota*, *Thermodesulfobacteriota*, *Actinomycetota*, and *Pseudomonadota* were observed. At the phylum level (Figure 11A), the YN group exhibited *Bacillota* and *Bacteroidota* at 83.9% and 1.02%, with a *Bacillota*/*Bacteroidota* (F/B) ratio of 82.25. In contrast, the AN group displayed proportions of 75.46% and 9.90% for *Bacillota* and *Bacteroidetes*, resulting in a reduced F/B ratio of 7.62 (*p* < 0.05). Post 2′-FL intervention, the Y 2′-FL group had *Bacillota* and *Bacteroidota* proportions of 85.04% and 0.99%, respectively, with an F/B ratio of 85.90, while the A 2′-FL group exhibited proportions of 80.28% and 3.66%, respectively, yielding an F/B ratio of 21.93. It was observed that 2′-FL could elevate F/B ratios in the Y 2′-FL group (*p* > 0.05) and the A 2′-FL group (*p* < 0.05). Furthermore, compared to the AN group, the proportions of *Bacillota* and *Bacteroidota* in the A FOS group were 61.98% and 6.98%, respectively, with an F/B ratio of 8.88, suggesting an elevated F/B ratio with FOS intervention in the A FOS group (*p* > 0.05). Overall, 2′-FL and FOS might enhance the gut microbiota structure in the naturally aging mouse model. Examining the genus-level variations among the treatment groups (Figure 11B and Figure 12E–H), following 2′-FL administration, the Y 2′-FL group displayed increased proportions of *Lachnospiraceae*_UCG-006 (*p* > 0.05) compared to the YN group; the A 2′-FL group displayed increased proportions of norank_f__ *Muribaculaceae* (*p* < 0.05) and *Lachnospiraceae*_NK4A136_group (*p* > 0.05) compared to the AN group. Similarly, post FOS intervention, the A FOS group exhibited noticeably elevated relative abundances of the *Akkermansia* genus (*p* < 0.05) in comparison to the AN group.

Through the application of LEfSe analysis, we aimed to identify distinctive gut microbiota biomarkers among the various experimental groups. Analysis in Figure 11C revealed that post-treatment with 2′-FL, the Y 2′-FL group exhibited an increased abundance of the genera *Lachnospiraceae*_UCG-006; the A 2′-FL group displayed elevated levels of norank_f__*Muribaculaceae* and *Lachnospiraceae*_NK4A136_group. Subsequent intervention with FOS led to a notable prevalence of the *Akkermansia* genus in the A FOS group, aligning with patterns observed in the genus-level bar graph in Figure 11B and Figure 12E–H. This observation suggested a specific association of *Lachnospiraceae*_UCG-006, norank_f__ *Muribaculaceae*, and *Lachnospiraceae*_NK4A136_group with the 2′-FL treatment, while *Akkermansia* stands out as a genus distinctively linked to the FOS intervention.

## 4. Discussion

This study employed C57BL/6J mice to confirm the regulatory role of 2′-fucosyllactose (2′-FL) in promoting intestinal health in aged mice. The results demonstrated that 2′-FL could synergistically enhance intestinal barrier function, suppress chronic inflammation, optimize gut microbiota composition, and further regulate immune homeostasis through microbial metabolites, offering novel theoretical and experimental insights into gut health intervention mechanisms for older people.

This study found that the administration of either FOS or 2′-FL had no adverse effects on the body weight or overall physiological condition of the mice, confirming the safety of 2′-FL as a functional dietary supplement. Dinleyici et al. [27] reported that the addition of HMOs to infant formula supports healthy development in infants, further confirming the safety of HMOs. Additionally, the present study found that 2′-FL significantly upregulated the expression of tight junction proteins (ZO-1 and Claudin-1), reduced intestinal permeability in aged mice, improved intestinal barrier function, and effectively suppressed the secretion of pro-inflammatory factors such as IL-6 and TNF-α. Ye et al. [28] reported that a prebiotic formula containing 2′-FL and FOS suppressed serum cytokine levels, such as IL-4 and IL-10, and upregulated the expression of tight junction proteins through the TLR-4/MAPK/NF-κB signaling pathway, thereby enhancing intestinal barrier function. Furthermore, Carter et al. [29] conducted a study in elderly individuals, confirming that long-term 2′-FL supplementation strengthened the intestinal mucosal barrier, reduced chronic inflammation, and significantly decreased inflammatory markers in certain individuals. These findings align with our results. Previous studies have highlighted that the gut microbiota and metabolic niches contribute to intestinal barrier integrity by enhancing epithelial defense mechanisms [30,31]. Consequently, we hypothesize that the improvement of intestinal barrier function and anti-inflammatory effects of 2′-FL in aged mice are closely associated with its modulation of the gut microbiota.

The present study revealed that 2′-FL significantly increased fecal water content and substantially decreased fecal pH in aged mice. It is acknowledged that alterations in gut microbiota composition closely correlate with fecal pH and hydration levels. Wong et al. [32] demonstrated that *Bifidobacterium*, known as a beneficial microorganism in infant intestinal flora, generated short-chain fatty acids (e.g., acetate and lactate) during metabolic processes, which were subsequently converted into butyrate. This metabolic pathway led to a notable reduction in fecal pH and strengthened the gut’s ability to resist colonization by harmful pathogens. Shan et al. [33] discovered that the combined action of 2′-FL and *Bifidobacterium bifidum* (*B. bifidum*) modulated the water absorption capacity of the intestines in mice by boosting the population of beneficial bacteria like *Akkermansia*, *Parabacteroides*, and *Bifidobacterium*. Furthermore, this cooperative effect enhanced the intestinal barrier’s functionality by mitigating inflammation, ultimately increasing fecal water content, enhancing defecation performance, and fostering gastrointestinal health.

This study utilized 16S rRNA sequencing to investigate the impact of 2′-FL on the gut microbiota in aged mice. The results demonstrated that 2′-FL increased the levels of beneficial bacteria, such as *Lachnospiraceae*_UCG-006, *Lachnospiraceae*_NK4A136_group and norank_f__*Muribaculaceae*, leading to enhanced microbial diversity and a decrease in pathogenic groups. Previous studies shown that the elevation of *Lachnospiraceae*_UCG-006 induced by a prebiotic diet was advantageous for gut health, as it correlated positively with γ-aminobutyric acid (GABA) and 5-hydroxyindole acetic acid (5-HIAA) [34]. Studies demonstrated that *Lachnospiraceae*_NK4A136 aided in digestion and the production of short-chain fatty acids (SCFAs), notably acetate, butyrate, and propionate, offering protective effects against harmful microorganism growth in the gut while promoting epithelial cell proliferation, thus enhancing intestinal barrier function and positively influencing gut health maintenance [35,36,37]. These outcomes further validate our research, affirming that 2′-FL could enhance the intestinal barrier through gut microbiota modulation.

Moreover, unlike conventional interventions that solely impact microbial abundance, the metabolic network affected by 2′-FL intervention might be more intricate, involving alterations in various signaling molecules like short-chain fatty acids and tryptophan derivatives. Wu et al. [38] revealed that 2′-FL and 3′-FL potentially boosted the relative levels of *Lactobacillus* and *Bifidobacterium*, leading to the production of short-chain fatty acids (acetate and lactate). This action reduced the pro-inflammatory cytokine IL-4 levels while increasing IFN-γ levels, thereby enhancing immune balance in allergic mice. Additionally, Wang et al. [39] indicated that after 2′-FL entered the intestinal tract, a portion of it could be utilized by the intestinal flora and was metabolized into small molecules such as short-chain fatty acids, stimulating the release of mucus, reducing the expression levels of TNF-α and IL-1β genes, and increasing the level of tight junction protein Claudin-1. Thus, it alleviated oxidative stress in the aging mouse model and exerted anti-inflammatory effects; the other portion interacted with intestinal epithelial cells, further enhancing gut integrity, mitigating mucosal inflammation, and bolstering the intestinal mucosal barrier. Furthermore, Yan et al. [40] confirmed that following 2′-FL intervention, the populations of *Bifidobacterium* and *Lactobacillus* increased in colonic mice suffering from colitis, leading to a rise in the content of the tryptophan derivative indole-3-lactic acid (ILA), increasing the level of anti-inflammatory factors (IL-10), thereby suppressing inflammatory responses. These studies implied that gut microbiota’s immune modulation might be linked to signaling pathways influenced by their metabolic byproducts, prompting our future in-depth exploration in this area.

Furthermore, this study found that 2′-FL significantly downregulated the expression of key cellular senescence markers, P16^INK4α^ and P21^Cip1^, in the aged gut. Combined with the results of gut barrier and inflammatory factors, we speculated that 2′-FL might alleviate the senescence-associated secretory phenotype [41], which can directly impair epithelial integrity and exacerbate local inflammation. This apparent anti-senescence effect was likely mediated indirectly by 2′-FL-induced alterations in the gut microbiota and its metabolites, such as short-chain fatty acids, which have been implicated in modulating host cellular aging pathways [42]. These findings suggested that 2′-FL might effectively reduce the accumulation of senescent cells in intestinal tissue, thereby alleviating cellular senescence in the intestinal epithelium. Senescent cells secrete pro-inflammatory factors, a phenomenon referred to as the senescence-associated secretory phenotype [43], which can directly impair epithelial integrity and exacerbate local inflammation. The observed reduction in P16^INK4α^ and P21^Cip1^ expression may, in turn, attenuate the senescence-associated secretory phenotype, which aligns with the improved intestinal barrier function and reduced inflammation noted in our study. Specifically, downregulation of P21^Cip1^ could influence cell cycle arrest following DNA damage [44,45], potentially enhancing the proliferative and tissue repair capacity of intestinal cells, a result consistent with our histological analysis. This apparent anti-senescence effect is likely mediated indirectly by 2′-FL-induced alterations in the gut microbiota and its metabolites, such as short-chain fatty acids, which have been implicated in modulating host cellular aging pathways [42]. Future studies investigating senolytic therapies are necessary to validate the mechanisms by which 2′-FL confers its gut health benefits, particularly through the clearance of senescent cells.

Additionally, it is important to highlight that young mice were utilized as a control group for comparison in this study. The research revealed that while 2′-FL also exhibited some beneficial effects on the intestinal health of young mice, its positive impact on the intestinal health of aged mice was more prominent. Specifically, when compared to the young mice, the older negative mice displayed relatively inferior levels in various metrics (such as fecal water content, pH value, cytokines, and tight junction proteins). This disparity can be attributed to the inherent age-related irregularities in the physiological conditions of the mice, particularly within the gastrointestinal system [46]. Rinninella et al. [6] also noted that aging led to the deterioration of the primary immune system and the decline of the epithelial barrier in the gastrointestinal tract, resulting in compromised intestinal health. Furthermore, it was observed that the influence of 2′-FL on the gut microbiota in aged mice was notably more profound. This difference was likely linked to the aging process, which caused alterations in digestion, nutrient absorption, and immune response, leading to a more uniform gut microbiota composition in aged individuals [47,48] which was verified by our study as well.

Additionally, this study revealed that 2′-FL tended to show stronger effects on the intestinal health of aged mice than FOS, though direct differences were not significant. This could be attributed to the distinctive structure of 2′-FL, enhancing its suitability as a substrate for beneficial bacteria and effectively activating immune-related pathways [49,50]. Zhang et al. [51] demonstrated that, in contrast to lactose and FOS, 2′-FL selectively boosted the growth of acid-producing bacteria and short-chain fatty acids in the small intestine and colon, leading to alterations in microbiota composition and metabolism. In contrast to FOS, while 2′-FL and 3′-FL effectively mitigated barrier dysfunction caused by IL-6 and symptoms of DSS-induced colitis, they bolstered the expression of tight junction proteins (ZO-1 and Claudin-1) to enhance intestinal barrier function. Furthermore, 2′-FL and 3′-FL enhanced the diversity of the intestinal microbiota community, thereby supporting gut equilibrium [52]. These results suggested that 2′-FL effectively improved intestinal health in aged mice; however, given the limited sample size, additional investigations with larger sample cohorts and independent replication will be conducted to further confirm the robustness and generalizability of these findings.

## 5. Conclusions

In conclusion, this study has demonstrated that 2′-FL effectively enhanced the physiological functions of intestinal health in aged mice by improving the intestinal barrier function, reducing inflammation, and regulating the gut microbiota. Nonetheless, animal models exhibited discrepancies with the complexity of the human body, including variations in baseline microbial diversity, immune–mucosal–metabolic interactions, and spatiotemporal kinetics. Moving forward, we will further explore the specific intervention mechanisms of 2′-FL in aged populations with diverse microbiomes using human samples, employ multi-omics methodologies, and conduct long-term monitoring to assess its clinical applicability, with the goal of establishing viable new strategies and targets for the prevention and management of age-related chronic intestinal conditions.

## Figures and Tables

**Table 1:** List of primer sequences for RT-qPCR.

Gene	Gene Sequence (5′-3′)	
ZO-1	M0098f	TCTGATGGTGCTCTGCCTAAT
	M0098r	GTCGCAAACCCACACTATCTC
Claudin-1	M0011f	TTATCGGAACTGTGGTAGAAC
	M0011r	CTCAGGGAAGATGGTAAGGTA
IL-1β	M0565f	AACAACAGTGGTCAGGACATA
	M0565r	GGGAAGGCATTAGAAACAG
IL-6	M0067bf	CGGAGAGGAGACTTCACAGAG
	M0067br	ATTTCCACGATTTCCCAGAG
P16^INK4α^	M1506bf	AAGAGCGGGGACATCAAG
	M1506br	CCAGCGGAACACAAAGAG
P21^Cip1^	M1359f	GGTTCCTTGCCACTTCTTAC
	M1359r	CTAACTGCCATCCCTGTTCTA

## Data Availability

The original contributions presented in the study are included in the article, further inquiries can be directed to the corresponding authors.
